# Stopping Onabotulinum Treatment after the First Two Cycles Might Not Be Justified: Results of a Real-life Monocentric Prospective Study in Chronic Migraine

**DOI:** 10.3389/fneur.2017.00655

**Published:** 2017-12-04

**Authors:** Paola Sarchielli, Michele Romoli, Ilenia Corbelli, Laura Bernetti, Angela Verzina, Elona Brahimi, Paolo Eusebi, Stefano Caproni, Paolo Calabresi

**Affiliations:** ^1^Neurology Clinic, University of Perugia—Perugia General Hospital, Perugia, Italy; ^2^Regional Health Authority, Public Health Regional Department, Perugia, Italy; ^3^IRCCS Santa Lucia, Rome, Italy

**Keywords:** chronic migraine, onabotulinum toxin A, botox, migraine, migraine treatment

## Abstract

**Introduction:**

Onabotulinum toxin A (OnabotA) cyclic treatment is approved for the prophylactic treatment of chronic migraine (CM), a highly disabling disorder. Although treatment response varies among patients, current guidelines suggest to stop treatment after cycle 2 if no response is achieved. This prospective study aimed to define, in real-life setting, the evolution of the response to OnabotA over five cycles of treatment among patients non-responding to cycle 1. The results of this study might help in decision-making, in particular whether prosecuting OnabotA further or not, when facing a patient not responding to cycle 1.

**Methods:**

Patients failing to respond at cycle 1 were recruited to complete five cycles. Key outcomes were: (i) a ≥50% reduction in headache days, (ii) a ≥50% reduction in total cumulative hours of headache on headache days and (iii) a ≥5-point improvement in Headache Impact Test-6 (HIT-6) scores.

**Results:**

Overall, 56 patients were included. Mean age was 45.7 years (female 83.9%). Severe (≥60) HIT-6 score was reported at baseline by 95.8% of patients. Responders (headache days reduction of more than 50%) progressively increased cycle after cycle, doubling from cycle 2 to cycle 5 (from 27 to 48%). In addition, patients regressed from CM to episodic migraine moving on with each cycle, with 78% of them reaching less than nine migraine days/month after cycle 5. The headache days per month decreased significantly from cycle 1 to cycle 5 (overall from 23.3 ± 5.7 to 9.2 ± 3.6; *p* < 0.001). During 12 months (5 cycles), migraine days per month progressively abated (from 18.5 to 8.7; *p* < 0.001), days with symptomatic medications intake/month consistently decreased (from 17.4 to 8.1; *p* < 0.001), and mean HIT-6 score lowered (from 72.4 ± 5.7 to 50.2 ± 4.3; *p* < 0.001).

**Conclusion:**

The positive effect of OnabotA treatment spreads over the course of the treatment and might also manifest late in treatment course among patients with no benefit after the first two cycles. Thus, the results of this real-life study suggest to extend OnabotA treatment further, beyond cycle 2, to avoid premature withdrawal in patients who would have become responders at cycle 3, 4, or 5.

## Introduction

Chronic migraine (CM) is the most disabling migraine headache disorder, involving 2% of the general population, with an estimated incidence of about 2.5% per year (1, 2). CM clinical scenario is defined by headache occurring on 15 or more days per month during more than 3 months, with migraine headache features on at least 8 days per month (3). CM has an outstanding impact on health-related quality of life, work productivity, and health-care resource utilization (4). Moreover, a remarkable proportion of patients overuse symptomatic medications, developing medication-overuse headache (MOH), thus needing detoxification and specific prophylactic treatments (3, 5). Despite the need for preventive therapy for CM patients, poor evidences exist on prophylaxis with oral drugs in CM (6).

Onabotulinum toxin A (OnabotA) is the only preventive treatment approved for CM based on efficacy and safety data from randomized controlled trials, with efficacy being also confirmed in patients with MOH (5, 7–10). OnabotA is typically given every 12 weeks, following the standard treatment PREEMPT protocol and dosage (7). From its approval, OnabotA efficacy and safety have been confirmed in real-life studies, with significant positive impact also on comorbid depression, anxiety, and health-related quality of life (11–16). Nevertheless, several questions are unanswered: whether patients not responding to the first cycle of treatment might respond to subsequent cycles is one of the most relevant.

Using pooled data from PREEMPT trial, Silberstein and colleagues defined the probability of non-responders to cycle 1 to improve after cycle 2 and 3. Specifically, more than 10% of patients were reported to respond to cycle 2 and 3 after having failed to respond to cycle 1 (late responders), suggesting an improvement in the short-term period with repeated treatment (17), as well as it was described in long-term follow-up (18). Nevertheless, current guidelines for OnabotA treatment in CM recommend to stop treatment if no benefit is achieved in two consecutive cycles (19).

To date, no studies are available, in real-life setting, regarding the proportion of patients non-responding to cycle 1 who first respond to OnabotA in the following cycles. Moreover, few data are available regarding the evolution of CM during the five cycles in the sub-population of non-responders to the first cycle of treatment. In this prospective real-life setting study, we report the evolution in response to OnabotA repeated cycles among patients non-responding to cycle 1. The results of this study might help clinicians in decision-making about continuing OnabotA treatment under such specific circumstances.

## Materials and Methods

### Study Design

This open-label, single-arm, prospective, observational study has been conducted in accordance with the principles of the Helsinki Declaration. The study was approved by the Internal Advisory Board. Participants admitted to the Headache Centre of Neurologic Clinic of Perugia between January 2014 and September 2016 were enrolled, after obtaining a written informed consent, according to the following inclusion criteria: (a) 18–65 years of age; (b) diagnosed with CM according to ICHD-III-beta criteria (3); (c) received and failed at least two other oral preventive drugs; (d) scheduled to receive OnabotA according to PREEMPT paradigm; (e) non-responders at cycle 1. Patients were not excluded in case of overuse of symptomatic drugs, in line with previous real-life setting studies (11–16). No prophylactic drug was prescribed or withdrawn. Eligibility was confirmed by a protocol-specific checklist. Seventy-one patients were selected according to specified criteria; only 56 accepted to prosecute OnabotA treatment over five cycles (1 year). No demographic differences were found between the groups. Study visits were programmed every 4 weeks. Patients recorded the days with migraine together with the characteristics of the attack and symptomatic drug consumption on a diary. OnabotA was administered at the Headache Center of Perugia, every 3 months (±1 week) for five cycles, following the PREEMPT injection paradigm, with OnabotA 155 U administered in 31 fixed-site, fixed-dose injection in seven specific head/neck muscle areas, and additional 40 U available for specific sites using a follow-the-pain strategy (7). No further doses were administered. Response was defined according to previously reported paradigms (16) as a ≥30% of reduction in headache days, according to headache diary, from the 4-week pretreatment to any of the three 4-week periods during treatment cycles. Among those who benefited from treatment, we divided partial responders (30–49% reduction) from responders (≥50% reduction). Patients experiencing reduction inferior to 30% were considered as non-responders. Beyond the number of headache days, defined as a 0–24 day with at least 4 h of headache, we also considered: (a) number of acute medication intake days and (b) Headache Impact Test (HIT)-6 scores, ranging from 36 to 49 to indicate little or no impact, from 50 to 55 to indicate some impact, from 56 to 59 to indicate substantial impact, ≥60 to indicate a severe impact. Key outcomes (Figure S1 in Supplementary Material) were: (i) a ≥50% reduction in moderate/severe headache days, (ii) a ≥50% reduction in total cumulative hours of headache on headache days, and (iii) a ≥5-point improvement in HIT-6 scores. Efficacy measures were collected at baseline (referring to the previous 3-month diary) and every 3 months at the time of each injection, and after 3 months from cycle 5. In case of reduction of headache days/month beyond 15, we divided headache frequency in three stages (0–4, 5–9, and 10–14 days/month) to check for evolution beneath the threshold of CM. Patients were given specific diaries to be filled out in the 3 months between cycles, to assess number and clinical features of headache attacks as well as symptomatic medication intake. Specific training was provided to ensure accurate completion of questionnaires. Results refer to the 3 months before the cycle of treatment, from which the benefit was obtained. As a safety measure, adverse events, related to the drug, were also registered at each time point.

### Statistical Analysis

Descriptive statistics are presented as means and SDs for continuous variables. The categorical variables were reported as counts and percentages. Mann–Whitney *U* test was used for testing hypotheses of changes of continuous variables. Fisher exact test was used for testing hypotheses of difference in the distribution of categorical data. A 0.05 level of significance was assumed for all the hypotheses tests.

## Results

Seventy-one patients were selected according to specified criteria; 56 accepted and were recruited to complete a five-cycle (1 year) OnabotA treatment period. Fifteen patients did not participate to the study: seven refused to undergo further invasive treatment, two refused to be included in the study but continued OnabotA cycles, and six refused to prosecute the treatment because of the lack of short-term guaranteed results. Non-participants had baseline characteristics similar to participants. Among participants, follow-the-pain strategy with up to 195 U of OnabotA was used in 59 cases (21.0%), due to shortening of response persistence. Demographic and headache characteristics at baseline and after cycle 1 are reported in Table 1. Mean age was 45.7 ± 6.5 years; female gender prevailed (79.7%). The mean time since migraine diagnosis was 14.2 (±5.1) years, while time since CM onset was 7.9 ± 4.3, ranging from 1 to 17 years. Severe (≥60) HIT-6 score was reported by 95.8% of patients, with 71.4% of them incurring in symptomatic medications overuse according to ICHD-3 beta definition (3). All patients failed at least two preventive medications, including topiramate. Previous ineffective medications ranged from 2 to 6 (2.58 ± 0.49). Baseline and 3 months after cycle 1 treatment data highlight the non-responsiveness of the whole cohort to cycle 1 treatment with OnabotA.

**Table 1:** Baseline demographic and clinical data (*n* = 56).

Mean age, years (range)	45.7 ± 6.5 (26–67)
Female % (*n*)	83.9% (47)
Years from migraine diagnosis (range)	14.2 ± 5.1 (5–25)
Years from CM diagnosis (range)	7.9 ± 4.3 (1–17)
Days with headache/month at baseline	23.1 ± 6.3
Days with headache/month after cycle 1	23.3 ± 5.7
Days with migraine/month at baseline	18.9 ± 5.6
Days with migraine/month after cycle 1	18.5 ± 4.3
Medication intake days per month at baseline	18.0 ± 4.4
Medication intake days per month after cycle 1	17.4 ± 3.6
HIT-6 score at baseline	72.1 ± 6.0
HIT-6 score after cycle 1	72.4 ± 5.7

*Data are presented as mean ± SD; Baseline data refers to the 3 months before starting Onabotulinum toxin A treatment*.

*CM, chronic migraine; HIT-6, Headache Impact Test-6*.

Variations of all outcome measures from baseline to OnabotA cycle 5 are reported in Table 2. The headache days per month decreased significantly during the 1-year treatment period from cycle 1 to cycle 5 (overall from 23.3 ± 5.7 to 9.2 ± 3.6; *p* < 0.001). Moreover, significant improvements in all other key outcomes were found. Migraine days per month progressively abated during the five cycles (from 18.5 ± 4.3 to 8.7 ± 2.5; *p* < 0.001), as well as days with acute medications intake (from 17.4 ± 3.6 to 8.1 ± 2.6; *p* < 0.001).

**Table 2:** Variations in outcome measures referred to the OnabotA cycles.

Time point	Headache days per month	Migraine days per month	Medication intake days per month	HIT-6
After cycle 1	23.3 ± 5.7	18.5 ± 4.3	17.4 ± 3.6	72.4 ± 5.7
After cycle 2	17.4 ± 4.7*	12.2 ± 4.7*	11.4 ± 3.6*	66.2 ± 5.1*
After cycle 3	12.6 ± 4.2*	10.5 ± 3.4*	10.3 ± 3.7*	57.5 ± 4.5*
After cycle 4	10.3 ± 3.2*	9.3 ± 2.7*	9.1 ± 3.2*	54.6 ± 5.3*
After cycle 5	9.2 ± 3.6*	8.7 ± 2.5*	8.1 ± 2.6*	50.2 ± 4.3*

*p < 0.001. Data are presented as mean ± SD.

*HIT-6, Headache Impact Test-6; OnabotA, onabotulinum toxin A*.

**Figure 1:**
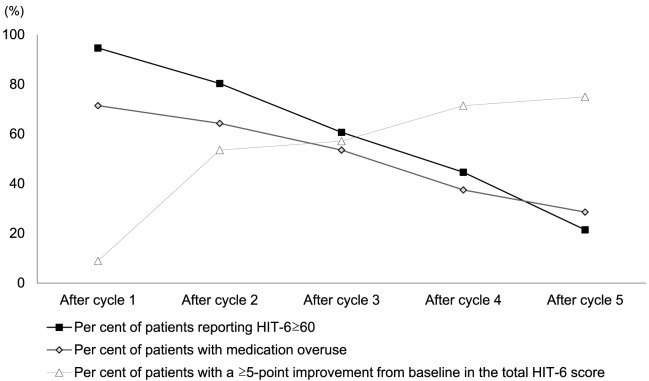
Evolution of Headache Impact Test-6 (HIT-6) scores and medication overuse with treatment cycles. Results are presented as percentages of patients with HIT-6 ≥ 60, patients with medication overuse and patients obtaining a reduction higher than 5 points from previous HIT-6 score. Fischer Exact test was used for testing distribution of data. Comparing values in each cycle with the previous cycle, significant improvement (*p* < 0.05) was found for all items considered. Raw data in Table S1 in Supplementary Material.

What is more, the mean HIT-6 score progressively decreased during the year of treatment (from 72.4 ± 5.7 to 50.2 ± 4.3; *p* < 0.001), with a severe impact on the rate of patients with severe (≥60) HIT-6 score, decreasing by nearly 60% moving from cycle 2 to cycle 5 (Figure 1; Table S1 in Supplementary Material). Medication overuse was significantly limited as well (71.4% after cycle 1 vs 28.6% after cycle 5). Symptomatic medication intake/day, ranging 0–6 at baseline, decreased to 0–3 after treatment completion.

**Figure 2:**
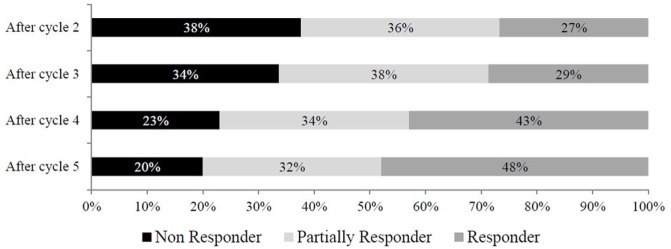
Evolution over treatment cycles of the response to onabotulinum toxin A treatment. Non responder: patients with a reduction of days with headache per month <30%; partially responder: patients with a reduction of days with headache per month between 30 and 49%; responder: patients with a reduction of days with headache per month ≥50%.

The proportion of non-responders to treatment dramatically decreased over time, reaching its half at the end of treatment protocol (Figure 2). On the contrary, partial responders and responders constantly increased with cycles of treatment, with responders almost doubling from cycle 2 to cycle 5 (27 vs 48%). Overall, summing up patients with a partial and optimal response, OnabotA treatment provided a significant benefit (≥30% reduction in headache days/month) in 80% of patients after cycle 5, with a 17% increase in response moving from cycle 2 to cycle 5 (from 63 to 80%). Interestingly, 18 patients who were non-responders after cycle 2 become responders (*n* = 14) or partial responders (*n* = 4) after cycle 5. Similarly, three non-responders and three partially responders after cycle 4 became responders at cycle 5.

**Figure 3:**
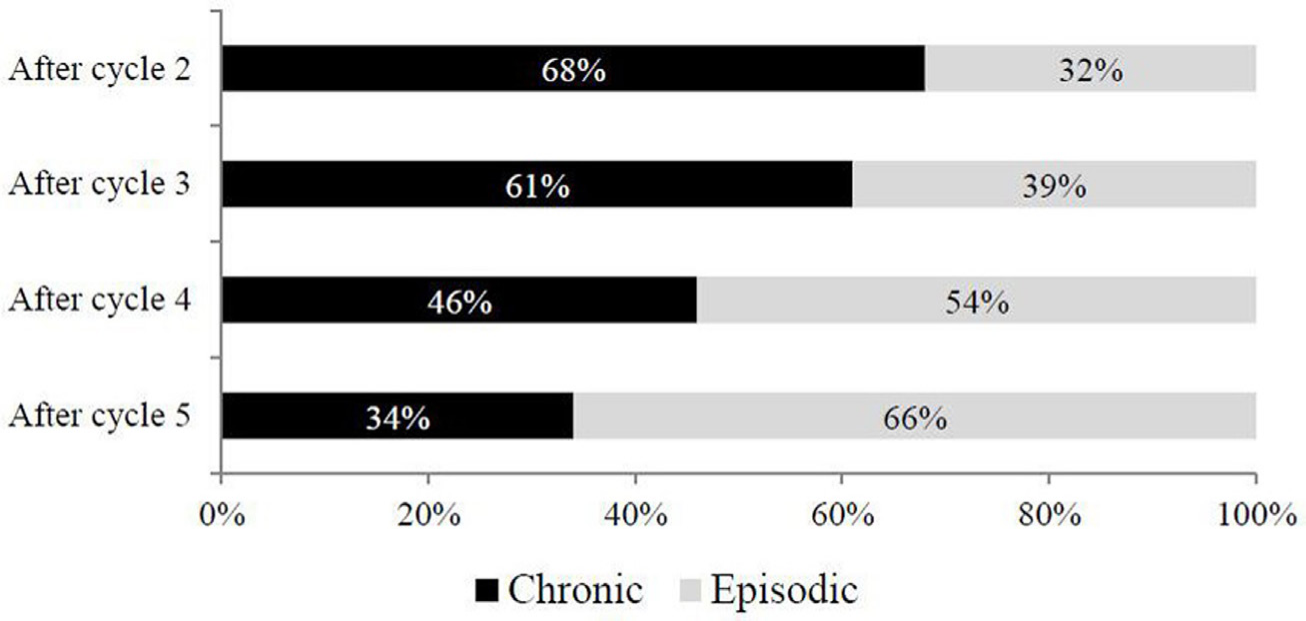
Conversion rate from chronic to episodic migraine (EM) across treatment cycles. Chronic: chronic migraine according to ICHD-III beta criteria (3); episodic: EM according to ICHD-III beta criteria (3).

A significant positive trend with treatment cycles was observed also for other outcome measures, such as migraine days per month (Figure 3). In particular, the proportion of patients moving from CM to episodic migraine (EM) doubled from cycle 2 to cycle 5 (32 vs 66%), while patients still experiencing CM radically decreased from 68 to 34% (Figure 3).

**Figure 4:**
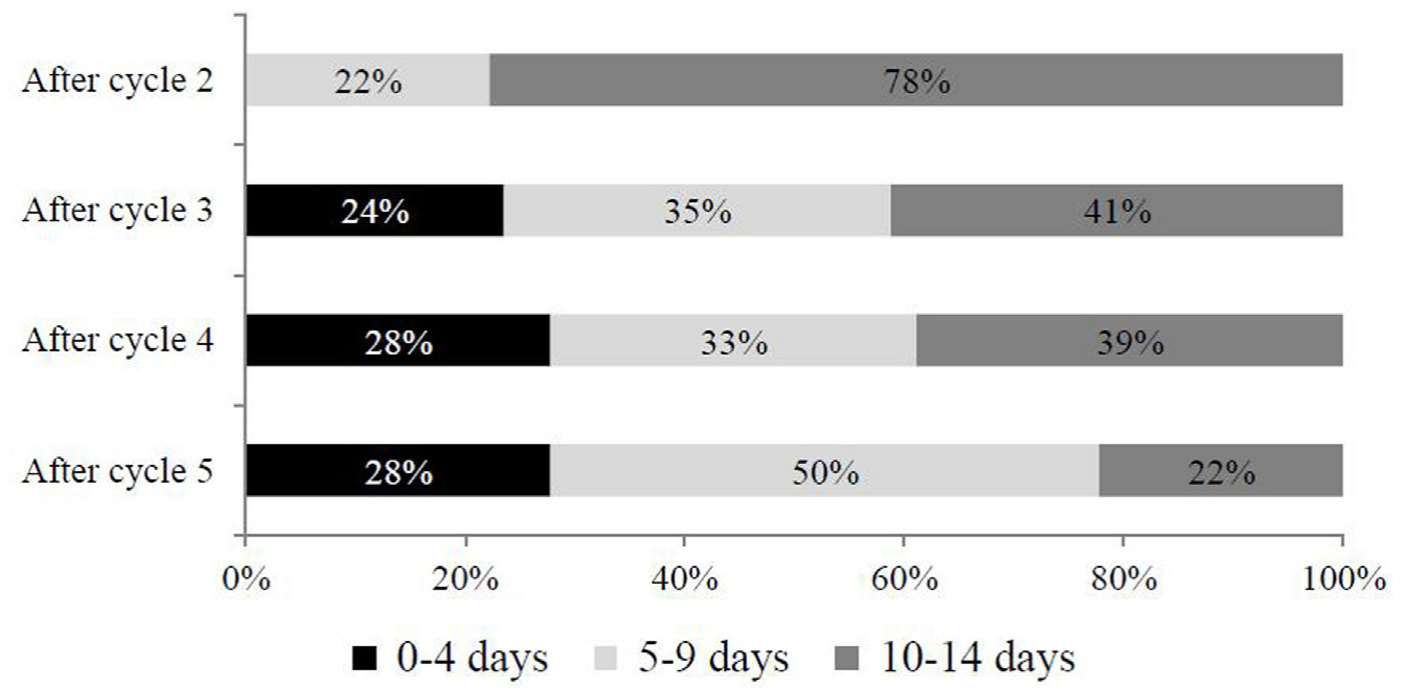
Evolution of migraine days per month during treatment among responders.

Considering different intervals of migraine days per month (0–4 vs 5–9 vs 10–14 days), OnabotA showed a positive progressive impact among late responders (Figure 4). Overall, patients having suboptimal response (10–14 days of headache/month) significantly decreased from 78% after cycle 2 to 22% after cycle 5. On the contrary, the proportion of patients achieving 5 to 9 days of headache/month progressively increased, moving from 22% at cycle 2 to 50% at cycle 5. What is more, treatment with OnabotA had a significant impact on achieving the target of 0–4 days of migraine/month, since after cycle 2 no patient achieved it, vs 28% of patients at cycle 5 (Figure 4).

Adverse events related to OnabotA treatment were consistent with the safety and tolerability profile of this drug among CM patients. In particular, 7% of patients reported mild to moderate adverse events, lasting less than 1 week, including pain in the site of injection, headache, and cervical musculoskeletal weakness. None of patients develop eyelid ptosis.

## Discussion

Onabotulinum toxin A efficacy among patients with CM has been widely shown in clinical trials and real-life setting (11–18). Guidelines for OnabotA treatment in CM recommend to stop treatment if no benefit is achieved in two consecutive cycles (19). However, a recent PREMPT *post hoc* analysis highlighted that at least 20% of patients non-responding to cycle 1 become responders at cycle 2 and 3, with significant benefit on quality of life (17). Thus, to continue or not to continue treatment after failure to respond to the first cycles is matter of debate. However, poor data exist on this issue, especially in the specific sub-population of non-responders to the cycle 1. In this prospective real-life setting study, a specific cohort has been selected: indeed, only patients failing to respond to cycle 1 OnabotA have been included. Assessing the evolution of response rate among such patients, we showed that benefit and progressive conversion to responder status is achieved within five treatment cycles. In particular, looking at the evolution of responder status beyond the first three cycles [the time limit of the PREEMPT *post hoc* analysis (17)], a further proportion of patients converts to responder status. Overall, in our real-life setting study, from cycle 2 to cycle 5 the proportion of responders raised by 20%, with full responders almost doubling, from 27 to 48%. What is more, non-responders were nearly halved moving from cycle 2 to cycle 5. Thus, conversion of non-responders can happen beside cycle 3, with substantial benefit on headache and quality of life. Moreover, once response is achieved among previously non-responders patients, a consistent reduction in headache days/month is observed, with significant conversion rate to EM status (66% at cycle 5), and high impact on quality of life, as shown by HIT-6 score decrease.

According to current migraine guidelines an adequate trial of preventive treatment is recommended before define the treatment ineffective (19). As far as OnabotA treatment is concerned, NICE recommendations suggested to stop OnabotA treatment in two cases: (1) if the number of days with migraine each month has not been reduced by at least 30% after two OnabotA cycles and (2) if headache days have been reduced under 15 days per month for a 3 months in a row (19). These recommendations have been fully embraced by national regional health system committees to design diagnostic and therapeutic guidelines. However, the results of this real-life setting study suggest that two treatment cycles might not be enough to assess ineffectiveness of OnabotA. On the contrary, it might be extended to at least 1 year, because a significant proportion of patients non-responding at the very beginning could actually benefit from OnabotA, becoming responders after the first 2, 3, or 4 cycles.

Moreover, regarding the interruption of OnabotA treatment after conversion from CM to EM, this prospective study highlights a progressive reduction in patients with 10 to 14 headache days/month (from 78% at cycle 2 to 22% at cycle 5). Since these patients with frequent EM are likely to progress to CM (20), one may argue that interrupting treatment cycles might increase the risk of relapse into CM. On the contrary, providing five OnabotA cycles might significantly reduce headache days per month well below 10, with significant impact on patients quality of life. Our cohort is still being followed-up to confirm net benefit over the second year of OnaobotA treatment.

Several factors are known to influence the response of CM to OnabotA, including peripheral and central sensitization mechanisms (17). OnabotA blocks neurotransmission *via* SNARE complex cleavage, inhibiting the release of CGRP, substance P and glutamate. Such effects, together with the regulation of the expression of the transient receptor potential vanilloid type 1, localized within C-fibers and participating in pain transmission, directly limit peripheral sensitization. Moreover, once peripheral sensitization is reduced, central sensitization indirectly decreases, leading do pain relief (21). All of the factors taking part to the effects of OnabotA on peripheral and central sensitization might also influence treatment response (21). This means that the response to OnabotA might be extremely subjective, and some patients might need repeated dosing to achieve headache control or resolution (17). This study shows that patients not responding to OnabotA cycle 1 might indeed benefit from further treatment cycles. Cumulative effects of OnabotA, with progressive remodeling of peripheral sensitization directly, and central sensitization indirectly (20, 21), might participate in the progression of response to treatment seen in this study. However, since CM represents a highly heterogeneous condition, with several factors leading to chronification, all variables must be taken into consideration to understand the complex functioning of prolonged OnabotA on CM physiopathology. Moreover, further studies are needed on the prediction of treatment response. To date, patients with higher CGRP levels have been shown to better respond to OnabotA treatment, suggesting that CGRP pathway modulation is an essential part of OnabotA effects among chronic migraineurs (22). Thus, to correctly tailor treatment to patients, clinicians might consider to prosecute OnabotA among non-responders at cycle 1 only if CGRP levels are increased, since those patients are likely to turn into late responders (22). Nevertheless, better response-predictive instruments are needed, which will eventually pave the way for an individualized patient-centered treatment. Larger cohorts trial might identify also clinical characteristics predictive of OnabotA response, which also in this study failed to be spotted. Concluding, the results of this study suggest that, to guarantee an effective therapeutic option, clinicians might consider the opportunity to prosecute OnabotA among patients not responding to the first cycles. Indeed, stopping treatment too early might hinder late responders to benefit from OnabotA, and increase the risk of regression from EM to CM and to the maladaptive status underlying central sensitization mechanisms. Better prediction of response to treatment might, in the near future, allow a further refinement of treatment paradigm.

## Ethics Statement

This observational study received approval from the Internal Advisory Board. Informed consent was obtained from all patients for data collection, analysis, and publication.

## Author Contributions

PS performed and took full responsibility for data collection and analysis, interpretation of data and results, manuscript drafting and revision. MR revised the manuscript. IC, LB, AV, EB, SC, and PC revised the manuscript. PE performed statistical analysis.

## Conflict of Interest Statement

The authors declare that the research was conducted in the absence of any commercial or financial relationships that could be construed as a potential conflict of interest.

## Abbreviations

CM, chronic migraine; EM, episodic migraine; HIT-6, Headache Impact Test-6; MOH, medication-overuse headache; OnabotA, onabotulinum toxin A.
