# Supplementary figures and images for: Stopping Onabotulinum Treatment after the First Two Cycles Might Not Be Justified: Results of a Real-life Monocentric Prospective Study in Chronic Migraine
Source: Front Neurol. 2017 Dec 4;8:655. doi: 10.3389/fneur.2017.00655 (PMC5723003; doi:10.3389/fneur.2017.00655)

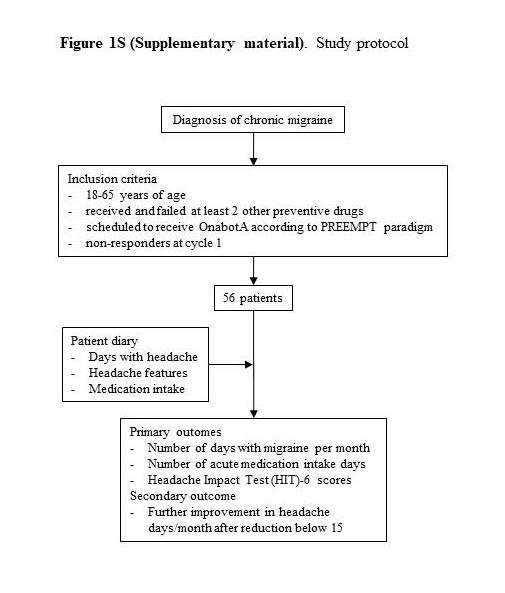

Supplement: Supplementary file 1 [file Image_1.jpeg]
