# Supplementary material for: Stopping Onabotulinum Treatment after the First Two Cycles Might Not Be Justified: Results of a Real-life Monocentric Prospective Study in Chronic Migraine
Source: Front Neurol. 2017 Dec 4;8:655. doi: 10.3389/fneur.2017.00655 (PMC5723003; doi:10.3389/fneur.2017.00655)
Supplement: Supplementary file 2 [file Table_1.PDF]

Table S1. Evolution of HIT-6 scores and medication overuse with treatment cycles

	**After cycle 1**	**After cycle 2**	**After cycle 3**	**After cycle 4**	**After cycle 5**
Per cent of patients reporting HIT-6≥60	53 (94.6%)	45 (80.4%)*	34 (60.7%)*	25 (44.6%)*	12 (21.4%)*
Per cent of patients with medication overuse	40 (71.4%)	36 (64.2%)*	30 (53.6%)*	21 (37.5%)*	16 (28.6%)*
Per cent of patients with a ≥5-point improvement from baseline in the total HIT-6 score	5 (8.9%)	30 (53.5%)*	32 (57.1%)*	40 (71.4%)*	42 (75.0%)*

Statistical analysis was performed to compare the distribution of variables with different cycles of treatment. *=p<0.05
